# Cellulose/Collagen Dressings for Diabetic Foot Ulcer: A Review

**DOI:** 10.3390/pharmaceutics12090881

**Published:** 2020-09-17

**Authors:** Ruth Naomi, Mh Busra Fauzi

**Affiliations:** Centre for Tissue Engineering and Regenerative Medicine, Faculty of Medicine, Universiti Kebangsaan Malaysia, Cheras, Kuala Lumpur 56000, Malaysia; ruthmanuel2104@gmail.com

**Keywords:** dressing, cellulose, collagen, diabetic foot ulcer, wound closure, rapid healing

## Abstract

Diabetic foot ulcer (DFU) is currently a global concern and it requires urgent attention, as the cost allocation by the government for DFU increases every year. This review was performed to provide scientific evidence on the advanced biomaterials that can be utilised as a first-line treatment for DFU patients. Cellulose/collagen dressings have a biological property on non-healing wounds, such as DFU. This review aims to analyse scientific-based evidence of cellulose/collagen dressing for DFU. It has been proven that the healing rate of cellulose/collagen dressing for DFU patients demonstrated a significant improvement in wound closure as compared to current standard or conventional dressings. It has been scientifically proven that cellulose/collagen dressing provides a positive effect on non-healing DFU. There is a high tendency for cellulose/collagen dressing to be used, as it highly promotes angiogenesis with a rapid re-epithelisation rate that has been proven effective in clinical trials.

## 1. Introduction

### 1.1. Diabetic Foot Ulcer 

**Figure 1:**
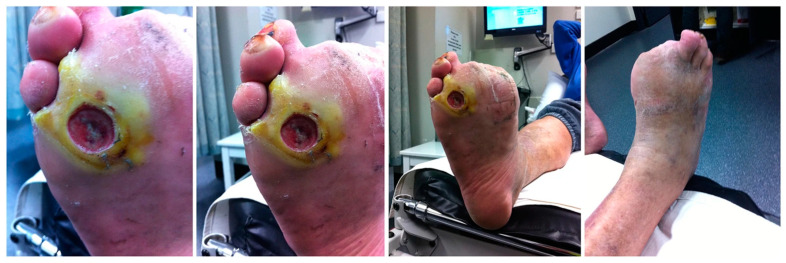
Diabetic Foot ulcer. Figure reused from Netten and co-workers [24]. Used under the creative. Commons license http://creativecommons.org/licenses/by/4.0/.

Diabetic Foot Ulcer (DFU) is a chronic condition that resulted from uncontrolled diabetes that leads to peripheral artery disease or neuropathy. A single or combination of both abnormalities will trigger the development of DFU. DFU usually leads to progressive bone, joint, and soft tissue deterioration with commonly seen in the ankle and foot [1], causing severe complications that inflict more than 50% of amputations in diabetic patients. This is mainly caused by impaired wound healing due to the thickening of the basement membrane, foreign body infection, low proliferation rate, irregular keratinocyte differentiation, and slow angiogenesis that perpetuates the foot deformity. In this case, amputation and surgical debridement is the only available treatment to prevent further systemic spread of infection [2,3]. Meanwhile, DFU has been categorised as a significant mortality determinant in a study that revealed half of the subjects who developed DFU tend to die within five years [4]. The global prevalence rate of DFU has been estimated to be around 6.3% and Asia recorded around 5.5%. There is a high possibility for amputation within the first year of infection, which is around 34.1% with a mortality rate of 5.5% [5]. Figure 1 shows the condition of DFU. 

### 1.2. Diabetic Foot Ulcer Scenario in Malaysia

Malayjsia has been ranked as the highest diabetes cases recorded in Asia and it is one of the highest in the world [6], with around 3.6 million patients [7]. By 2025, it is expected that around seven million adults will suffer from diabetes and potentially to develop DFU. The prevalence rate was identified to be approximately 31.3% for adults above 18 years old [7]. According to the wound care unit at Hospital Kuala Lumpur (HKL), 70% of the patients are diabetic patients who are suffering from foot ulcers and more than 12,000 patients have visited the wound care unit since 2013. Diabetic patients may take up to three months to completely heal with proper care and this is four times higher when compared to the normal healing rate [8]. According to the data that were collected from the general hospitals in Malaysia (2009), there is a notable increase in patients with foot complications from 1549 subjects screened for diabetics. From this, 5% demonstrated an absence of foot pulse, 3.9% with a healed ulcer, 3.8% patients with leg amputations, 2% subjects who undergone angioplasty or vascular surgery, while 1.5% with an active ulcer or gangrene. The majority of about 27% patients have been confirmed with foot infection, while the remaining patients have been diagnosed with infection at various sites in the body, particularly in the urinary tract, skin, respiratory tract, and eye [9].

### 1.3. Socioeconomic Burden

Globally, it is estimated that £252 million was allocated for DFU treatment annually. This excludes the indirect cost due to the loss of productivity, quality of life, and family status. The total expenditure for the management of diabetic foot ulcer ranged from US$9 billion to US$13 billion in a year. A minor limb amputation costs around US$43,800, whereas a major amputation can cost up to US$66,215. This cost excludes rehabilitation care and medications [10]. In Malaysia, 13% of the total healthcare budget has been allocated for the management of diabetic patients in 2011, including societal expenditure, which can reach up to MYR3.52 billion per year [7]. In 2014, the Malaysian government allocated approximately MYR30 billion (USD6.8 billion) for the clinical sector in which diabetic foot infection remains a major burden [11].

### 1.4. Current Treatment for Diabetic Foot Ulcer

Total contact cast (TCC) is an offloading device providing mechanical support in treating DFU patients and it is currently the gold standard treatment [12,13]. It acts by redistributing the pressure in the plantar surface to the body mass as well as maintaining the subject’s mobility, which prevents the breakdown of new skin [14]. TCC is also known to be an affordable ambulatory technique [15] to assist many diabetic patients. Although the use of TCC provides several benefits, it leads to immobilisation and user’s safety is a major concern, as it requires skillful clinicians to apply it efficiently. Nevertheless, there is a notable difficulty when inspecting the wounds due to the distinctive obstacles faced during removing the attached device [12,13]. 

Other treatments, such as negative pressure wound therapy (NPWT), dermapace system (DS), and maggot debridement therapy (MDT), are the currently available treatments that have been used widely to treat DFU patients. NPWT is an existing modality to draw out the exudates from the ulcerated area through a pressure produced by a vacuum [16,17]. Upon extracting the excess fluid, this device creates a moisture microenvironment triggering the blood flow to the ulcerated region by removing wound healing inhibitors. This situation stimulates the formation of granulation tissue that induces stress at the wound bed and directly elevated the proliferation of the cell. This process contributes to wound healing [17,18]. 

DS utilises shock wave energy that resembles sound waves to the surface of the wound, which triggers the body’s wound healing pathway [19,20]. This device accelerates the process of angiogenesis that increases the production of growth factor (GF). Meanwhile, the transmitted wave assists in reducing inflammation that exists in the wound bed as well as to the tissues surrounding the wound. The application of this device to DFU resulted in notable wound closure [21]. MDT is also one of the current treatment options available for treating DFU. This therapy works by removing dead tissues that are present in the wound area using larva [20,22,23]. In this procedure, artificial and sterile grown larvae known as *Phaenicia sericata/Lucilia sericata* will be applied to the wound area to disinfect the wound region [22,24]. The larvae will remove the dead-tissue and release proteolytic enzyme to the wound region, which will then lyse the non-viable tissues in the ulcerated region contributing to the process of wound closure [25].

### 1.5. Contraindication and Complications of Current Available Treatment

Few issues need to be taken into consideration when using TCC. It is not advisable to apply TCC in patients with osteomyelitis, deep abscess, or gangrene. Extra care is also needed when applying TCC in unsteady gait patients. In certain case, there is a high possibility of skin breakage at the surrounding area of the existing ulcer if TCC is applied for a long period or have a sensitive skin [14]. Although, over time, the ulcers tend to have an improvement with TCC, it still takes over a year to completely heal especially for a chronic ulcer. This is because chronic ulcer requires frequent dressing of at least every two weeks. It could contribute to the high cost and affects the socioeconomic background of the patients [13,14,26,27]. Nevertheless, there are high risks of hazards for TCC applications, including gait instability, iatrogenic ulcers due to the discrepancy in the length of the limbs, falls [26], stiffness of the joints, atrophy of the muscle, and new ulcer formation [15]. The use of TCC is a “force compliance” making the patients less active compared to other available off-loading devices. This results in reduced vertical force in the foot, which may lead to posture instability. TCC application could result in iatrogenic infection or skin abrasion and the application of this cast for a long duration will result in muscle atrophy and deterioration in the density of the bone [27].

The application of NPWT in DFU patients for a long period with lack of proper care, such as fixing it too tight, might result in other complications. A recent retrospective case study, including 57 DFU patients treated with NPWT complications showed 49% of the patients developed maceration of skin at the borders of the wound, 14% of bleeding, 12% of necrosis at the wound area, 7% of developed systemic signs or infection, while 2% with severe pain during changing the dressing [28]. This therapy has been known as an expensive treatment as compared to the currently available conventional DFU treatment [29]. 

**Figure 2:**
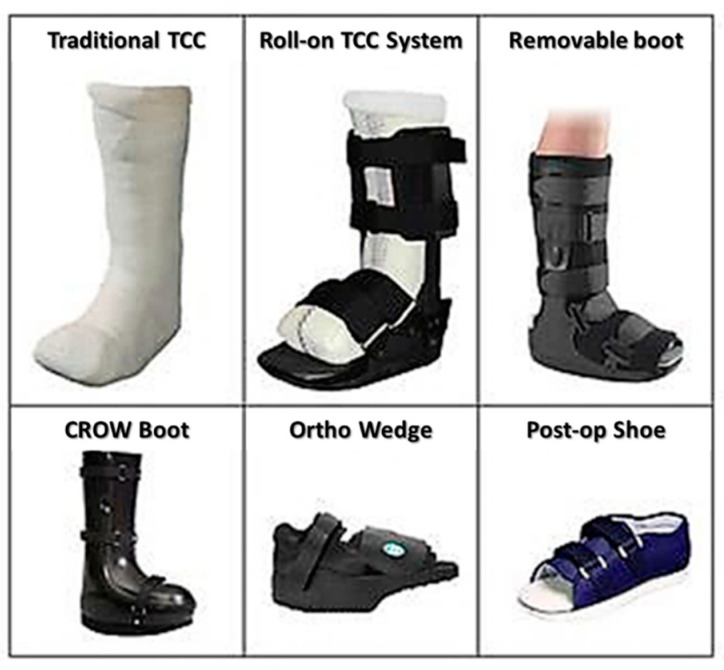
Current available treatment for Diabetic Foot Ulcer (DFU) [32]. Used under the attributions of creative. Commons license http://creativecommons.org/licenses/by/4.0/.

In contrast, even though DS provides several benefits, the disadvantage of this device outweighs its advantages. The advantages include the loss of sensation, local bruising, dizziness, nausea, infection in the wound site or beyond the wound, fever [19,20], and migraine [19]. The utilisation of MDT for DFU seems to be less effective, as it does not reduce the load of bacteria or improving the wound healing rate but only reduce the duration to debride the ulcerated region. At the same time, this therapy does induce anxiety among the patients as well as lead to the formation of erythema due to the digestive enzyme that is released by the maggots during the therapy session [30,31]. Figure 2 shows the current available treatment for DFU. 

## 2. Collagen

**Figure 3:**
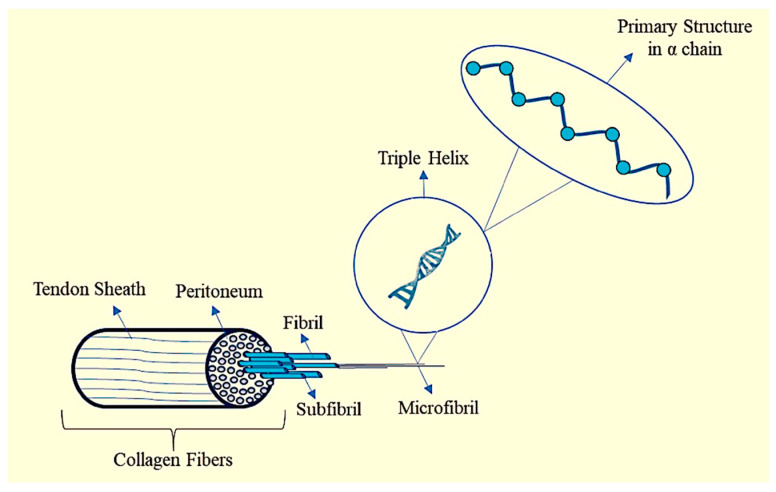
Structure of collagen.

Collagen is a natural fibrous protein in the body that makes up the connective tissues [33,34] in various sites. There about 28 different types of collagen that have been further classified according to their distribution and structure [35]. Collagen is a biocompatible structural protein, less immunogenicity, biodegradable, and biomimetic, which makes it an ideal source of biological materials for tissue engineering and regenerative medicine. The composition and functionality of the collagen fibres influence the cellular response that is commonly regulated by integrin. This phenomenon is achieved by a biological process that is known as fibrillogenesis [36]. Fibrillogenesis is a process of collagen network formation and interaction within the cellular level to form higher-order three-dimensional structure. Usually, these fibres are stabilised through a cross-linking intervention after a collagen scaffold formation to sustain its bioactivity and bioavailability [37,38]. By modifying each phase of fabrication based on the specific need of the tissues, bioscaffolds can be customised in order to improve its therapeutic effects [39]. Meanwhile, cross-linking can be achieved through a synthetic or natural polymer [40]. Genipin, oxidised alginate, dialdehyde starch, and procyanidin are natural-based crosslinkers [41], whereas synthetic crosslinker includes actin [42], polylactic acid, poly ahydroxyesters, and polyl glycolic acid [43]. Generally, crosslink molecules are equipped with two or more reactive ends that have the capacity to bind with the triple helix structure of the collagen. The crosslink can either appear inside or between the microfibre of the collagen. The microfibrils interdigitate and cross-link, thereby intactly avoiding detachment from one another [44]. Collagen can be extracted through chemical or enzymatic hydrolysis approaches. The extraction process was initiated with the removal of the inter- and intra-molecular bond. It was first pre-treated with alkaline (NaOH) or acid solution to expel non-collagenous substances. In chemical-based extraction of collagen, the pre-treated material commonly will be added with an acid-based solvent, such as 0.5 M of acetic acid, and stored up to 72 h. The precipitation step with salt (NaCI) solution until the supernatant becomes visible is crucial to allow the protein to bind prior to dialyse for two days with distilled water (constantly changed every 12 h). Otherwise, 0.5 M of acetic acid will be combined with enzymes, such as flavourzyme, alcalase, or pepsin, in the enzymatic collagen extraction. The solution will be continuously stirred at 4 °C up to 48 h, followed by filtration and dialysed for two days with distilled water to obtain the purified collagen [45]. Although chemical extraction is efficient [45], it is highly corrosive [46]. Meanwhile, enzymatic extraction of collagen requires a long duration to complete and it has a high possibility to contain incomplete hydrolysis process [46]; however, this method produces fewer waste products [45]. Figure 3 shows the structure of collagen.

## 3. Collagen-Based Treatment for DFU

Diabetes mellitus (DM) is one of the major concerns worldwide with DFU and is the most common complication of DM. A variety of advanced technology has been widely invented to cater this critical issue, especially collagen, which is known to be used the most. Collagen type I (Col-I) is deem required to attract GFs towards the wound site and to initiate wound healing and tissue regeneration. However, in the DFU case, the epidermis is ulcerated, leading to the disruption of the extracellular matrix contributing to tissue integrity loss resulting in Col-I deficiency. Besides that, it hinders the normal proliferation and migration of fibroblasts to the wound area and eventually slows down the wound healing [47]. Scientifically, it was proven that collagen accelerates wound healing and enhances re-epithelisation [48,49]. A study that was conducted by Ulrich and co-workers (2011) [43] recorded 32 DFU outpatients were successfully managed with oxidised regenerated cellulose (ORC) or collagen matrix. These patients who underwent this intervention show inhibition of protease, plasmin, and elastase, the most common causes of wound healing interruption. The excess level of elastase will interfere with the mechanism of normal collagen production, whereas plasmin has a significant role in hemolysis. These proteins (protease, plasmin, and elastase) distinctively act to degrade the fibronectin, endogenous protease inhibitors, and GF that are the essential elements in the wound healing process. In contrast, the intervention with ORC or collagen matrix at the specified wound region demonstrated an acceleration in ulcer healing on the 28th day as well as providing an optimum microenvironment for wound healing [48]. 

Meanwhile, a pilot study was performed on 22 patients with neuropathic DFU in order to evaluate the efficacy of wound dressing in wound closure with the intervention of equine pericardium collagen dressing. The dressing was consecutively changed every three to four days and in the fourth week, 94% of the patients showed a notable improvement in the wound region with 13% of complete heal, and both described a significant reduction in the ulcerated region (*p* < 0.0001). These findings indicated that collagen-derived dressing is safe and effective in accelerating ulcers healing [50]. A similar study was done on 124 chronic neuropathic DFU patients with formulated collagen gel (FCG), which comprise 2.6% of bovine collagen and adenovirus serotype 5 (Ad5). Upon implanting FCG at the wound site, 35% of ulcer closure has been identified on the 12th week. It showed that FCG enhances the expression of PDGF in order to accelerate wound healing. The presence of collagen in this product was proven to create a compatible and stable microenvironment at the injury site. This phenomenon was undertaken by holding the Ad5 at the ulcerated region by promoting the attraction via electrostatic force for GF to bind and encourage the formation of granulation tissue [51]. 

Furthermore, a comparative case-control study that was performed by Munish and co-workers (2015) described a significantly enhanced ulcer healing after being treated with collagen-based dressing [47]. The effectiveness of the treatment was evaluated through a study that was conducted on 25 patients with chronic DFU. The weekly assessment of the wound region was successfully performed from the first week of treatment until the 12th week. The 1st week of assessment identified two subjects that were completely healed and 12 subjects that showed a significant reduction in the size of the ulcer. Furthermore, the 12th week assessment showed 21 patients with completely healed and four patients with ulcer reduction. The enhancement of stimulation and differentiation of erythroid, granulocytes, and megakaryocyte precursor cells confirmed to increase defence mechanism. Because collagen biomaterial is slowly biodegraded; thus, it can act as a provisional bio-template for cell attachment, migration, and proliferation, as well as rapid wound maturation [52,53]. 

In contrast, the application of collagen powders to the ulcerated area create a more active site that allowed the fibronectin-binding as well as showing an increased in fibroblasts viability that plays the main role to accelerate wound healing [47]. Sprinkling collagen particles to the ulcerated region has been proven to decrease wound area with an absence of allergic response [53] and potentially to promote faster wound closure [54]. This is possible due to the ability of the collagen to retain its triple helix structure that preserved the thermal stability, mechanical strength, and functionality. This powdered collagen enhanced biomolecules interaction and the formation of a better three-dimension bio-scaffolding for cell migration prior to granulation tissue formation [46]. Additionally, it helps to hinder protease activity without affecting the performance and behaviour of GF [55]. On top of that, collagen can shrink the thickness of the scar, as it can regulate the collagenase activity and decomposition of the extracellular matrix through keratinocyte differentiation. Hence, collagen is assumed to be responsible for the scar size reduction as well as shorten the period of re-epithelisation [56].

## 4. Cellulose

**Figure 4:**
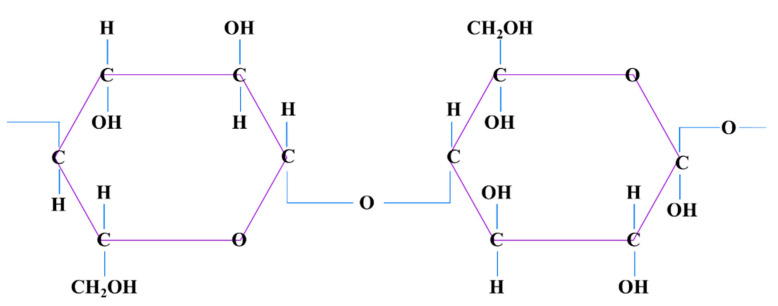
Cellulose structure.

Generally, cellulose is well known for its abundance of availability from plant sources. Cellulose is known to poses a strong intermolecular bond, which makes it impossible to dissolve in most of the solvents. Meanwhile, in a liquid state, cellulose exhibits great stability, optical, and mechanical characteristics [32,33], and, due to its ability to retain moisture, it can expedite the wound healing mechanism [34]. Generally, cellulose can be classified into natural and synthetic. Natural cellulose is derived from vascular plants, animals, or minerals. Synthetic cellulose is man-made modified cellulose from natural cellulose source [57,58]. Natural and synthetic cellulose are both bound together by intra- and inter-hydrogen bonds. Plant cellulose usually contains impurities such as lignin and hemicellulose whereas bacterial cellulose appears pure by nature. This makes the plant cellulose possess less crystalline property when compared to the bacterial cellulose [59]. Figure 4 shows the structure of cellulose. 

## 5. Cellulose as a Protective Barrier 

Cellulose can provide front-line protection against foreign material, including all types of cellulose derivatives. The bacterial cellulose (BC) is originated from a membrane of biosynthetic microfibrillar cellulose formed by Acetobacter Xylinum or other types of selected bacteria [60]. The bacteria are a non-harmful pathogen that possess the ability to provide a moisture surface to improve the debridement process and to expedite the re-epithelisation in chronic ulcers. In a wet state, the membrane of the cellulose has 84–89% crystallinity property, mechanical strength, and the capability to absorb water [61,62], which gives the capacity to shorten the duration of healing phases [61]. Besides that, BC has been scientifically proven to be biocompatible, biodegradable, non-toxic, and exceptionally pure that chemically resemble cellulose-derived from plants. Thus, BC has shown the intrinsic features that are suitable for a bio-scaffold as a protective barrier for skin injury in various form such as wound dressing [37]. 

Nevertheless, exudates may slow down the process of healing as it makes the tissue to separate from the wound region. When considering this, any wound dressing must be designed to reach the ability to absorb, retain and release liquid to achieve an optimum balance of wound healing microenvironment. Thus, BC stands as a defence mechanism through the pore size surface area and the presence of hydrophilic compounds that supports the water holding and release capability as well as assists in the elimination of exudates from the wound site [37]. An in vitro study that was conducted by Loh et al., (2018) showed that BC-incorporated hydrogel can maintain the viability of the cell and promote cell attachment [63,64]. This is due to the BC property, which forms a tight barrier between the environment and the wound. This serves as a protective compound against bacterial infection that will accelerate wound healing rate as compared to the negative control in an in vitro study [65]. The application of bacterial cellulose also showed a 75% reduction in the wound region in a non-healing lower extremity ulcer [66]. 

A recent study conducted by Pinho and co-workers (2018) presented that cotton cellulose functionalised with hydrogels can function as a carrier to deliver drugs through the hydrogel. This framework allows for the bioactive chemicals, painkillers, antibiotics, and other therapeutic substances to be transferred to the wound area. When this happens, the trapped molecules migrate from the polymeric network to the wound bed through a sustained release cycle [67]. This is done by switching the position with the exudate, thereby eliminating the exudates from the wounded site. This, in turn, serves as a physical shield against microorganisms being deposited and proliferated on the wound surface [68]. 

Seratica and co-workers (2010) stated that, with the presence of a unique characteristic of cellulose, partial-thickness wound healed better with MC dressing. A 100% healing rate on the fifth day in an in vivo model has been reported. Clinical testing involving 13 subjects showed a complete heal from a venous ulcer on the eighth week upon applying cellulose-based wound dressing [55]. This was made possible as MC possesses a greater impact with a greater tensile strength, crystallinity, and water absorption capacity compared to the plant cellulose. Plant cellulose provides a semi-permeable membrane at the wound region to improve angiogenesis and fibrinolysis [68,69].

In addition, a study showed a complete ulcer healing in a diabetic-induced mouse treated with nano bio-composite ointments comprise of bamboo cellulose within the 18th day and there was a notable deposition of collagen and regeneration of tissue at the wound region. The study confirmed that cellulose derived from plants incorporated with silver nanoparticles has anti-microbial characteristics and it can accelerate acute wound healing, as the test results showed an effective absorption of exudates, exchange of gas, and biocompatibility at the wound site [70,71]. Besides, the exploration of hybrid cellulose/collagen dressing for DFU is the alternative approach in wound healing management as demonstrated in Table 1.

## 6. Cellulose/Collagen Dressing for DFU

Collagen that is readily available in human skin mainly functions as an extracellular matrix that acts as a three-dimensional scaffold in the body microstructure [73]. This scenario explains the main reason for human body comprises up to 70–80% of collagen and various types of proteins. Six different types of collagen are present in the human body, specifically existing in the human skin and collagen type I particularly makes up 70% of the human skin composition [74]. Generally, the existence of collagen is essential to stimulate the migration of fibroblasts during injuries in which at later stage increase the deposition of secreted collagen at the wound bed to accelerate wound healing [75,76]. It has always been observed through newly formed skin at the wound site. On the contrary, the imbalance of collagen synthesis and degradation, especially in diabetic patient’s skin, commonly exhibited severe stiffness with poor flexibility [77]. In addition, the inflammatory phases of wound healing in diabetic patients were prolonged and the deposition of granulation tissue was hindered, leading to a slow phase in wound healing [78,79]. In a worst-case scenario, DFU patients commonly presented with a high blood glucose level in the body will have altered blood circulation. This hyperglycaemic condition induces oxidative stress on the nerve cells, leading to nerve damage, which triggers a condition known as neuropathy. This scenario explains the diabetic patients lose their sensation mostly in their limbs and unaware of developed blister or ulcers at the later stage. Over time, the optimum balance of collagen metabolism (production and degradation) was lost due to the prolonged conditions with the additional appearance of the ulcerated epidermis [80,81]. When considering this issue, collagen-based wound dressing was developed and introduced in order to accomplish the demand of the current therapeutic needs in wound management system [78]. Following that, the use of collagen is scientifically proven in assisting wound healing process from an in vivo model until the clinical trial stage. 

Manizate and co-workers (2012) conducted a comparative and prospective clinical study in 10 DFU patients, in which particular wounds were applied with either a sodium carboxy-methylcellulose or bovine native collagen dressing. The result demonstrated that bovine native collagen dressing that was incorporated with silver ion exhibited a normal activity of fibroblasts and protect the GF from being affected by the presence of matrix metalloproteinases (MMPs). In addition, it provides a moist environment to accelerate wound healing and also absorbs excessive exudates [69]. This finding was supported by Rangaraj and co-workers (2011), who stated that collagen dressing hindered the MMPs activity [74]. MMPs originated from natural endopeptidase that was frequently secreted by the normal cells, such as lymphocyte, granulocytes, and activated macrophage, into the extracellular matrix. A high level of MMPs will disrupt the interaction between GF and the extracellular matrix of the skin. The imbalance of MMPs and particular regulators will lead to an excess level of degradation activity. This causes a severe loss of extracellular matrix, thus slowing the re-epithelisation process during wound healing [81]. From the study conducted by Rangaraj and co-workers (2011), it has been proven that collagen balances the level of MMPs during an injury [74].

Another randomised control study was conducted by Gottrup and co-workers (2013) to evaluate the effect of oxidised regenerated cellulose (ORC) or collagen matrix as compared to the existing standard treatment. In this study, 39 DFU patients were chosen randomly and were divided into two groups, namely ORC dressing or collagen matrix and standard dressing comprising of 24 and 15 patients, respectively. By the fourth week, 50% of wound closure was observed among the subjects with ORC dressing. Weekly assessment at the 14th week unravelled a significant improvement in wound healing. Additionally, 52% of the subjects were completely healed by ORC dressing on the 14th week. No notable adverse effects or infection and a reduction in elastase were observed in patients receiving ORC treatments [62]. Elastase is a peptidase that is also a factor contributing to anti-healing by interfering with the synthesis of the collagen [48] and it can activate MMPs by degrading the existing connective tissue [82]. Thus, collagen dressing can act as a substrate for elastase activity that reduces the activity of elastase. Collagen tends to bind with elastase and this binding will not only alter the activity of elastase, but significantly improve wound closure. 

A similar study was conducted by Ulrich and co-workers (2011), studying the effect of ORC or collagen matrix on the level of plasmin, gelatinase, and elastase in wound exudate of DFU patients. The study revealed a significant reduction in the above-measured enzyme, together with MMPs and the wound size. Plasmin originated from serine protease family has the potential to turn on the MMPs in the extracellular matrix. This accelerates the degradation of fibronectin, protease inhibitors, and GFs. These factors are essential elements in wound healing, whereas the imbalance of gelatinase concentration affected the level of collagen type IV degradation [48]. This phenomenon explained the rapid wound closure within a short period of time, as collagen has the potential to regulate the level of protease enzyme in an exudate. Similarly, a study that was conducted by Griffin and co-workers (2019) proved that the early application of ORC or collagen matrix dressing has the potential to increase the formation of granulation tissue [69]. This later will be transformed into matured connective tissue during the remodelling phase to restore the tissue function [83]. The study described that ORC dressing for DFU patients has 82% effectiveness for the complete healing of ulcer as compared to ovine collagen extracellular matrix dressing with 15.2% worse condition on the existing ulcer upon application of the dressing to the affected region [71].

A study was done by Dumont and co-workers (2018), using a tridimensional collagen-based matrix, GBT013, a collagen-based dressing applied to the DFU patients proved that collagen has the ability to deteriorate MMPs and increases cell proliferation rate. It has been demonstrated to have more than 44% reduction in the ulcerated area of non-healing ulcers [72]. A randomised and prospective study on protease-modulated ORC dressing or collagen matrix was performed by Kloeters and co-workers (2015) for treating pressure on sore ulcers. The subjects receiving ORC or collagen matrix treatment showed a positive healing rate with a drastic reduction of plasmin level from the fifth until 28th day and elastase from the fifth day with 100% absence of intolerant towards the treatment and infection. Through this study, it has been proven that low level of plasmin activity accelerates angiogenesis by increasing the level of VEGF that plays a pivotal role in wound healing [64]. This finding has been further supported by Tahergorabi and co-workers (2012), revealing that VEGF has a higher potential to mediate abnormal angiogenesis [81]. 

Besides that, Solway and co-workers (2011), through their parallel open-label trial study, proved that microbial cellulose (MC) dressing for DFU patients enhances re-epithelisation and rapid wound closure in a short period of time. This is possible due to the presence of a microporous structure of the MC, which initiates the coagulation process by trapping platelets to stop the bleeding at the ulcerated area. Therefore, the MC acts as a temporary scaffold supporting the activity of keratinocytes, fibroblasts, and endothelial that endures the formation of granulation tissue, leading to rapid re-epithelisation process. By this, a conducive microenvironment with moisture-retaining capacity is created to enhance the wound healing phases [68]. Similar speculation has been offered by the MC to accelerate wound healing process from the inflammatory phase until the proliferative phase by initiating tissue regeneration, neuro-vascularisation, and cell differentiation. Nevertheless, MC triggers fibroblasts activity by attracting the ability of fibroblasts to infiltrate the wound site. Therefore, the increment of collagen deposition would enhance rapid wound healing through wound contraction mechanism [84,85].

Besides that, a recent study that was performed by Li and co-workers (2020) using a hyperbranched cationic polysaccharide-derived bacterial cellulose (BC) encapsulated with small interfering RNA (siRNA) was tested on a diabetic-induced mice model. The study described that BC dressing reduces MMPs level by releasing siMMP-9. At the same time, in vitro testing showed that siMMP-9 has less impact on cell membrane integrity, considerably low level of cytotoxic, and no keratinocyte cell death has been identified. This characteristic of BC incorporated with siRNA has been proven to expedite wound healing rate than that of diabetic-induced mice [72]. The finding was supported by Song and co-workers (2018), who revealed the effect of selenium-loaded cellulose film on a diabetic-induced rat model. The experiment proved that the tested biomaterial has an excellent microporous structure with high tensile strength. These properties contributed to the rapid contraction of the wound, thus accelerating the rate of wound healing as compared to the control. In addition, there was a notable increase in angiogenesis with matured blood vessels in diabetic rats [63].

Meanwhile, fabricated carboxymethyl cellulose (CC) incorporated with Ag-ZnO tested on in vitro model showed a permeable surface that is necessary for the formation of the tissue as well as bactericidal property towards Staphylococcus aureus and Escherichia coli. Nevertheless, this biomaterial exhibits greater swelling characteristics that are capable of absorbing liquid from wound exudate and supporting cell viability, especially the fibroblasts as the main player in the wound healing process. This fabricated biomaterial has been tested in vivo resulted in rapid wound healing with faster re-epithelisation and advanced development of extracellular matrices [86]. These results simply indicate that CC incorporated with Ag-ZnO is a hybrid biomaterial executed both healing property and a protective mechanism by acting as an antibacterial agent towards gram-negative and gram-positive bacteria. This was further clarified by Basu (2018), where the group unravelled that CC provides a conducive microenvironment favouring granulation tissue. This happens with the presence of polysaccharides to stop the bleeding at the wound region and CC absorbs the liquid from the wound exudate through ion exchange. Thus, the formation of granulation tissue and newly formed tissue will decrease the duration of the wound healing process. This situation occurs following the degradation of the polymer at the injury site that has been proven to stimulate the aggregation of the inflammatory cells, followed by fibroblast and epithelial cells migration [83]. Consequently, there will be rapid healing with notable wound closure at the site of the injury with the intervention of CC [87,88].

## 7. Synergistic Effect of Cellulose/Collagen Dressing 

**Figure 5:**
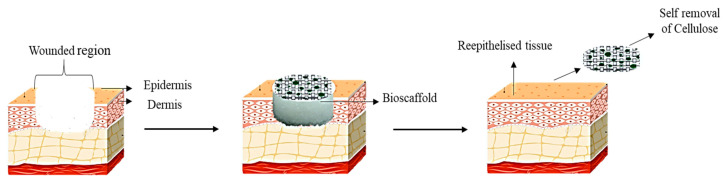
Mechanism of action of cellulose/collagen in ulcerated region.

Through electrospinning, cellulose acetate-collagen can be fabricated, and this fabricated biomaterial showed an abundance of mesenchymal cell proliferation on the scaffold indicating the capability to be used as wound dressing [31]. An in vitro study conducted by Vatankhah and co-workers (2013) showed that fibroblasts attached to the electrospun nanofibres after seven days. This indicates the capability of collagen secretion due to the high affinity of the cell to the scaffold [85]. This positive outcome of in vivo study serves as the main reason for the electrospinning cellulose acetate being proposed to be used in wound dressing. Another study shows that the hybridization of collagen or chondroitin sulfate incorporated with keratinocytes and sodium carboxymethyl cellulose incorporated with fibroblasts showed a compact stratified surface layer resembling epidermis. This indicates that the hybridisation of these scaffolds is a perfect biomaterial to be used as wound dressing [33] and hybridisation of cellulose and collagen enhances in vitro proliferation of fibroblasts [34]. Therefore, collagen presents an essential key factor in providing biological and structural integrity resembling native ECM. It is a complex system in which related substances undergoing continual remodelling to regulate the activity of the cell and tissue function. Furthermore, collagen is known as surface-active and is capable of breaching the lipid-free interface [35] and, upon placing the collagen scaffold at the ulcerated region, more fibroblasts will be attracted to the wound region to accelerate the normal healing pathway [36]. Meanwhile, cellulose is capable of absorbing exudate from the injured tissue, retaining moisture microenvironment, and accelerating the granulation tissue formation [37]. Thus, cellulose/collagen dressing is expected to accelerate the ulcers healing process by dual functions through the presence of collagen and cellulose, in order to attract fibroblasts and absorb the exudates from the injured tissue, respectively. An absence of any foreign materials will eventually contribute to a rapid healing mechanism. Over time, the collagen scaffold will be absorbed in the wounded region as it is highly biodegradable. In contrast, cellulose will fall off together with the scab as cellulose is not degraded by the human body. Figure 5 shows the possible mechanism of action of the cellulose/collagen in diabetic foot ulcer patients.

## 8. Advantages of Cellulose/Collagen Dressings

Cellulose/collagen dressing is reported to have benefits over the currently available conventional dressing in the market. Despite any sources of derivative of cellulose, it is proven to be highly biocompatible to clinical applications. However, cellulose obviously cannot be digested in the human body due to the lack of enzymes for breaking down the beta acetal linkages. Apparently, cellulose can dry and fall off together with wound scab over time upon healing completion. Meantime, collagen is compatible with humans due to its amino acid structure (R-G-D). As so, cellulose/collagen provides more beneficial effects in comparison to other dressings. Table 2 shows the advantages of cellulose/collagen dressing when compared to conventional dressing. 

## 9. Conclusions

This review summarises the positive effect of collagen as an advanced treatment for DFU patients as compared to cellulose, which is the most suitable to be used as a protective barrier due to its antibacterial characteristics. The hybridisation of collagen and cellulose is proven to enhance wound healing with rapid re-epithelisation and newly formed tissue. Further studies are needed in order to examine the mechanism of action for the hybridisation of collagen/cellulose dressing for DFU following the current high demand.

## Figures and Tables

**Table 1:** Cellulose/collagen dressing for DFU.

Author	Aim	Study Design	Sample Size	Follow Up	Findings	Conclusion
Manizate and co-workers (2012) [55]	Compare the efficiency of bovine native collagen with silver ion (Ag) and sodium carboxymethylcellulose with Ag	Comparative, post-market clinical evaluation	10 patients	1st and 4th week	-50% of the wound exhibit *S. Aureus* -By the 4th week, the bacteria load increases up to 1.53 × 10^5^ ppm	-Bovine native collagen dressing shows rapid wound closure.
Gottrup and co-workers (2013) [68]	Compare collagen/oxidised regenerated cellulose (ORC)/silver therapy to standard treatment	Randomized control trial	39 patients	Every 2 week for 14 weeks	-Decreased concentration of elastase, MMP-9 -low MMP-9:TIMP-1 concentration -Absence of infection and adverse effects	-Collagen/ORC/silver therapy shows improved wound healing.
Ulrich and co-workers (2011) [43]	Evaluate the effect of collagen matrix/oxidized regenerated cellulose in wound exudate of DFU patients	Comparative clinical study	32 patients	14th, 28th, 42th, and 56th day	-Reduced level of MMP-2 -Reduction in plasmin, elastase and gelatinase	-Wound size reduction on 14th and 28th days in ORC treated groups.
Griffin and co-workers (2019) [69]	Comparative study between the effectiveness of oxidized regenerated cellulose and ovine collagen extracellular matrix	Comparative study	3230 patients	4th, 8th, 12th, and 16th week	-82% of the healed wound with ORC dressing -15.2% of a worsened wound with ovine collagen extracellular matrix dressing	-ORC decreases healing duration by improving granulation tissue formation in a short period.
Dumont and co-workers (2018) [61]	Evaluate the effectiveness of collagen-based dressing for DFU patients	Clinical follow-up	6 male & 1 female	38th to 64th day	-Increased formation of granulation tissue -complete surface healing at the wound site	-Fast skin restoration -Decreased healing tine. -Decreased rate of infection.
Kloeters and co-workers (2015) [62]	Evaluate the effectiveness of oxidized regenerated collagen-cellulose matrix in pressure ulcer	Clinical assessment	33 patients	Weekly for 12 weeks	-Decreased level of plasmin and elastase activity -reduction in the surface area of the wound -Absence of infection and intolerance towards oxidized regenerated cellulose/collagen matrix dressing.	-Notable fast healing rate.
Solway and co-workers (2011) [65]	Study the effectiveness of microbial cellulose in DFU	Parallel open-label trial	34 patients	Weekly till complete wound closure	-Increased formation of granulation tissue and maintenance of moist environment at the wound area -High tensile strength and crystallinity of the microbial cellulose	-Rapid wound healing with a short period of re epithelisation
Li and co-workers (2020) [72]	Access the efficiency of naturally occurring bacterial cellulose-hyper branched cationic polysaccharide derivative on wound healing of diabetic rats	In vivo study	Not specified	1st, 4th and 7th day	-Good viability of cell -Low concentration of LDH -No effect on apoptosis -Inhibition in MMP-9	-Increased wound healing rate.
Song and co-workers (2018) [63]	Evaluate the effect of Selenium-loaded cellulose film in diabetic induced rats	In vivo study	48 male rats	3rd and 12th day	-Low elongation, high tensile strength, excellent microporous structure and high-water absorption capacity -Absence of toxicity	-Notable rapid wound healing. -Notable stimulation in the angiogenesis pathway.
Li and co-workers (2020) [64]	Evaluate the effectiveness of carboxymethyl cellulose/K-carrageena/graphene oxide/konjac glucomannan hydrogel in diabetic induced mice	In vitro and in vivo study	18 mice	4th, 7th, 14th and 21st day	-The presence of permeable surface, high mechanical strength and great swelling capacity, supports the viability of the cell and has bactericidal property.	-Notable rapid wound recuperating. -Advanced fibroblast production and rapid re-epithelialization were seen.

**Table 2:** Advantage of cellulose/collagen dressing.

Cellulose/Collagen Dressing	Conventional Dressing
Reduced reactive oxygen species in the wound [89]	Slow granulation tissue deposition [90]
Ability to absorb wound exudates [59]	High possibility for pathogenic organism to harbor [90]
Accelerates wound healing/promote rapid healing [91]	Dry, so it’s impossible to retain moist microenvironment [92]
Reduced length of stay in hospital [52]	Loss efficiency when loaded with absorbed wound exudates [93]
Shortened course of treatment [94]	Often requires extra care and frequent changing [95]
Improvement in wound reduction area [96]	
Rapid granulation tissue formation [90]	
Improved re-epithelisation and GF concentration [88]	
Absence/reduced bacterial invasion at the wound site [97]	
Cost effective [89]	
Easy application and good adherence to the wound bed [98]	

## Abbreviations

DFU	Diabetic foot ulcer
TCC	Total Contact Cast
NPWT	Negative Pressure wound therapy
AM	Amniotic membrane
DM	Diabetes mellitus
ORC	oxidized regenerated cellulose
GF	growth factor
FCG	formulated collagen gel
BC	bacterial cellulose
MC	Microbial cellulose
siRNA	small interfering RNA
CC	carboxymethyl cellulose
